# Lack of Association between Bax Promoter (-248G>A) Single Nucleotide Polymorphism and Susceptibility towards Cancer: Evidence from a Meta-Analysis

**DOI:** 10.1371/journal.pone.0077534

**Published:** 2013-10-17

**Authors:** Sushil Kumar Sahu, Tathagata Choudhuri

**Affiliations:** 1 Division of Infectious Disease Biology, Institute of Life Sciences, Bhubaneswar, India; 2 Department of Biotechnology, Siksha Vhabana, Visva Bharati, Santiniketan, Bolpur, India; National Center for Cell Science, India

## Abstract

**Background:**

The Bcl-2-associated X protein (Bax) is a proapoptotic member of the Bcl-2 family known to be activated and upregulated during apoptosis. Single nucleotide polymorphisms (SNPs) in Bax promoter may participate in the process of carcinogenesis by altering its own expression and the cancer related genes. Bax-248G>A polymorphism has been implicated to alter the risk of cancer, but the listed results are inconsistent and inconclusive. In the present study, we performed a meta-analysis to systematically summarize the possible association of this polymorphism with the risk of cancer.

**Methodology:**

We conducted a search of case-control studies on the associations of Bax-248G>A polymorphism with susceptibility to cancer in Pub Med, Science Direct, Wiley Online Library and hand search. Data from all eligible studies based on some key search terms, inclusion and exclusion criteria were extracted for this meta-analysis. Hardy-Weinberg equilibrium (HWE) in controls, power calculation, heterogeneity analysis, Begg’s funnel plot, Egger’s linear regression test, forest plot and sensitivity analysis were performed in the present study.

**Results:**

Cancer risk associated with Bax-248G>A polymorphism was estimated by pooled odds ratios (ORs) and 95% confidence intervals (95% CIs). The pooled ORs were calculated in allele contrast, homozygous comparison, heterozygous comparison, dominant and recessive model. Statistical significance was checked through Z and p-value in forest plot. A total of seven independent studies including 1772 cases and 1708 controls were included in our meta-analysis. Our results showed that neither allele frequency nor genotype distributions of this polymorphism were associated with risk for cancer in any of the genetic model. Furthermore, Egger’s test did not show any substantial evidence of publication bias.

**Conclusions/Significance:**

This meta-analysis suggests that the Bax-248G>A polymorphism is not an important cancer risk factor. Nevertheless, additional well-designed studies with larger sample size focusing on different ethnicities and cancer types are required to further validate the results.

## Introduction

The specific causes of cancer are not yet known. However, epidemiological studies contribute to suggest the relationship of genetic polymorphism to cancer susceptibility. Genetic association studies with single nucleotide polymorphisms (SNPs) targeting cancer are emerging area of research. SNP can be defined as a genomic locus where two or more alternative bases occur with substantial frequency of greater than 1% [1]. A total of around 10 million SNPs has been distributed throughout the human genome at a frequency of at least one in 1000 base pairs and exhibit low mutation rate [2]. Previous studies have shown that SNPs in oncogenes (e.g. Ras [3], mdm2 [4] and VEGF [5]) and tumor suppressor genes (e.g. p53 [6], Rb, and p16 [7]) may protect or increase susceptibility towards cancer risk. Thus, SNP analysis can be a tool for explaining the genetic complexities responsible for the cancer development to some extent. It promises to assist in the identification of novel cancer-related gene as a bio-marker for predicting an individual’s likelihood of developing cancer. The Bax gene is a well-studied tumor suppressor gene in various cell types. Bax is encoded by six exons and expresses a complex pattern of alternative RNA splicing that forms a 21kd membrane (α) and two forms of cytosolic protein (β and γ). It belongs to a proapoptotic member of the Bcl-2 family and has been implicated in the induction of apoptosis [8]. It remains inactive in cytosol of non-apoptotic cells during homeostatic conditions. Upon a cell death signal, Bax undergoes a conformational change that leads to their insertion, oligomerization and formation of large pores through the outer mitochondrial membrane [9]. Many apoptogenic factors like cytochrome c [10,11], Smack/Diablo [12], Omi/HtrA2 [13], endonuclease G [14] and apoptosis inducing factor [15] are released through this pore and recruit various molecules of apoptotic pathways. Numerous studies have demonstrated that alternation of Bax expression plays an important role in pathogenesis of cancer [16–21]. Genetic change of the Bax gene may have distinguished significance in cancer initiation and progression since it has a series of target genes including many oncogenes and tumor suppressor genes [22–26]. Studies on the association between SNPs in Bax and human cancer have provided new insights into the molecular mechanisms of cancer development. To date, at least 111 Bax SNPs have been reported in Enterzdatabase. In case of gastrointestinal cancer missense mutations of the Bax gene in codon 169 (Thr > Ala or Thr > Met) causes inhibition of the proapoptotic activity of the protein and enhance the development of cancer [27]. A guanine substituting adenosine at position 125 (G125A) in the Bax promoter is associated with higher stages of chronic lymphocytic leukemia (CLL) and failure to treatment response [28]. A silent point mutation in Bax codon 184 (TCG > TCA) has been reported in lung cancer patient [29]. Although Bax gene polymorphism has been suggesting its potential involvement in cancer development, current knowledge of the SNP located within the 5’-untranslated region of the promoter of the Bax gene, 248G>A, in cancer is still dispersive and limited. Bax-248GA polymorphism has been associated with down-regulation of Bax gene expression, advanced disease stage, lower treatment response and decreased survival rate in CLL and lung cancer. Also, there are contradictory reports showing lack of association between this polymorphism with risk of CLL and breast cancer. Thus, some group of researchers reported Bax-248GA polymorphism could be the bio-marker of susceptibility or protectibility to cancer [30–32], while other [33–37] shows a lack of association. However, with relatively small sample sizes, these former studies provided limited information and could not draw a convincible conclusion. Therefore, we performed a meta-analysis with all available studies (Checklist S1. PRISMA checklist.) to provide a more reliable conclusion about the relationship between Bax-248GA polymorphism and cancer risk.

## Materials and Methods

### Publication Search

**Figure 1:**
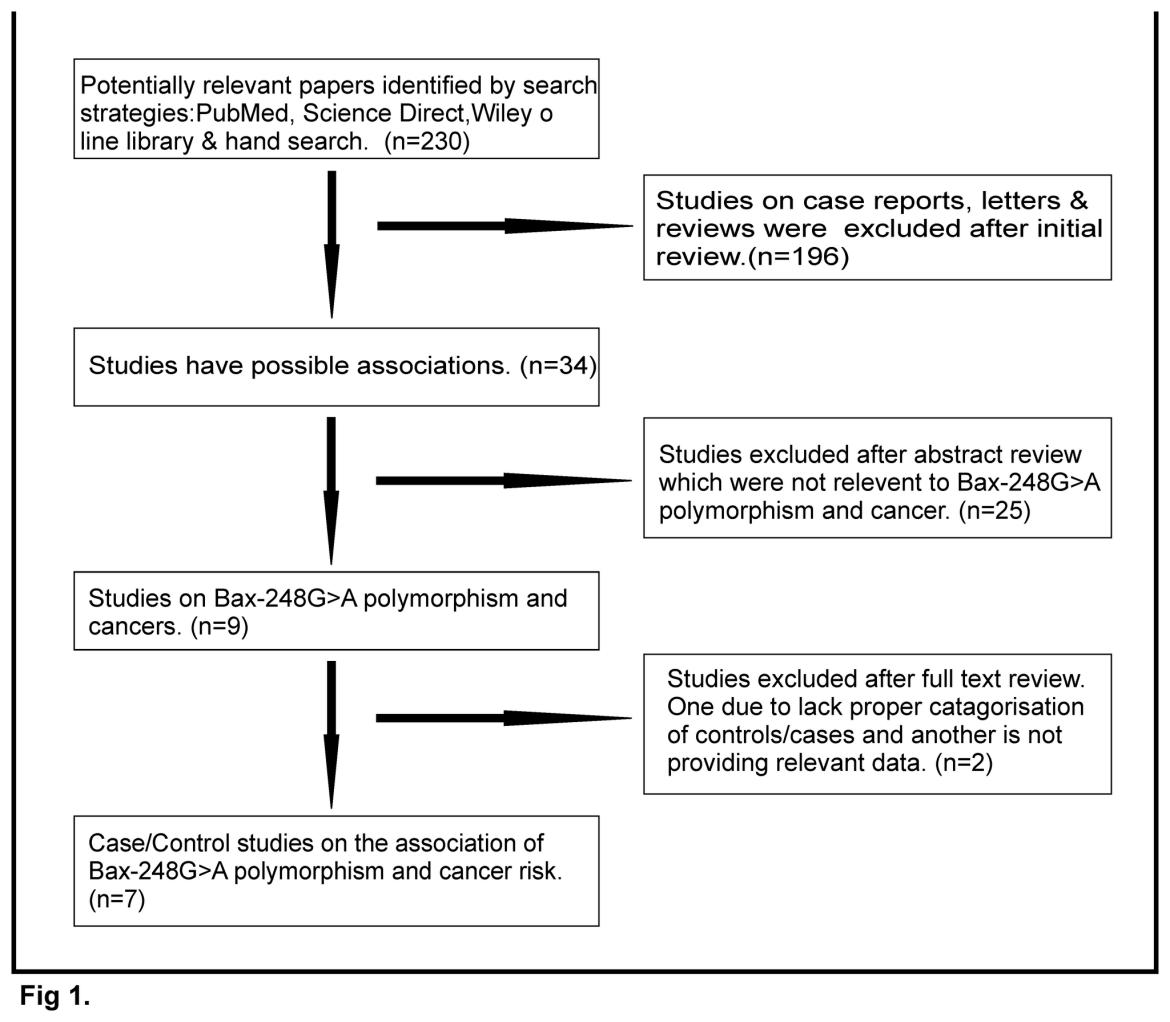
Flow diagram of articles selection. This is based on publication search, inclusion and exclusion criteria.

An extensive search was performed on Pub Med, Science Direct, Wiley Online Library and hand search with a combination of following search terms: Bax, polymorphism, SNP, mutation and/or cancer up to March, 2013. We evaluated all associated publications to identify the most eligible literature. The selection procedure of this study has been shown in Figure 1. The results were limited to papers published in English. Finally, seven studies were identified as eligible articles in this meta-analysis for Bax-248GA polymorphism, including four studies on CLL (812 cases and 462 controls), one study on squamous cell carcinoma (814 cases and 934 controls), one study on lung cancer (93 cases 230 controls) and one study on breast cancer (53 cases 82 controls).

### Inclusion and Exclusion Criteria

The following criteria were used to select studies for this meta-analysis (a) published in peer reviewed journals, (b) articles about Bax-248GA polymorphism and risk of cancers and (c) articles containing useful allele and genotype frequency. The exclusion criteria were (a) not case-control studies, (b) control population including with cases of cancers, and (c) duplication of a previous publication.

### Data Extraction

Two investigators (SKS and TC) reviewed and extracted the information from all eligible publications independently from each other according to the inclusion and exclusion criteria listed above. The following characteristics were collected from each study: first author’s name, year of publication, country of origin, cancer type, genotyping method for the assessment of Bax-248GA polymorphism, total number of controls and cases with G/G, G/A and A/A genotypes.

### Statistical Analysis

**Figure 2:**
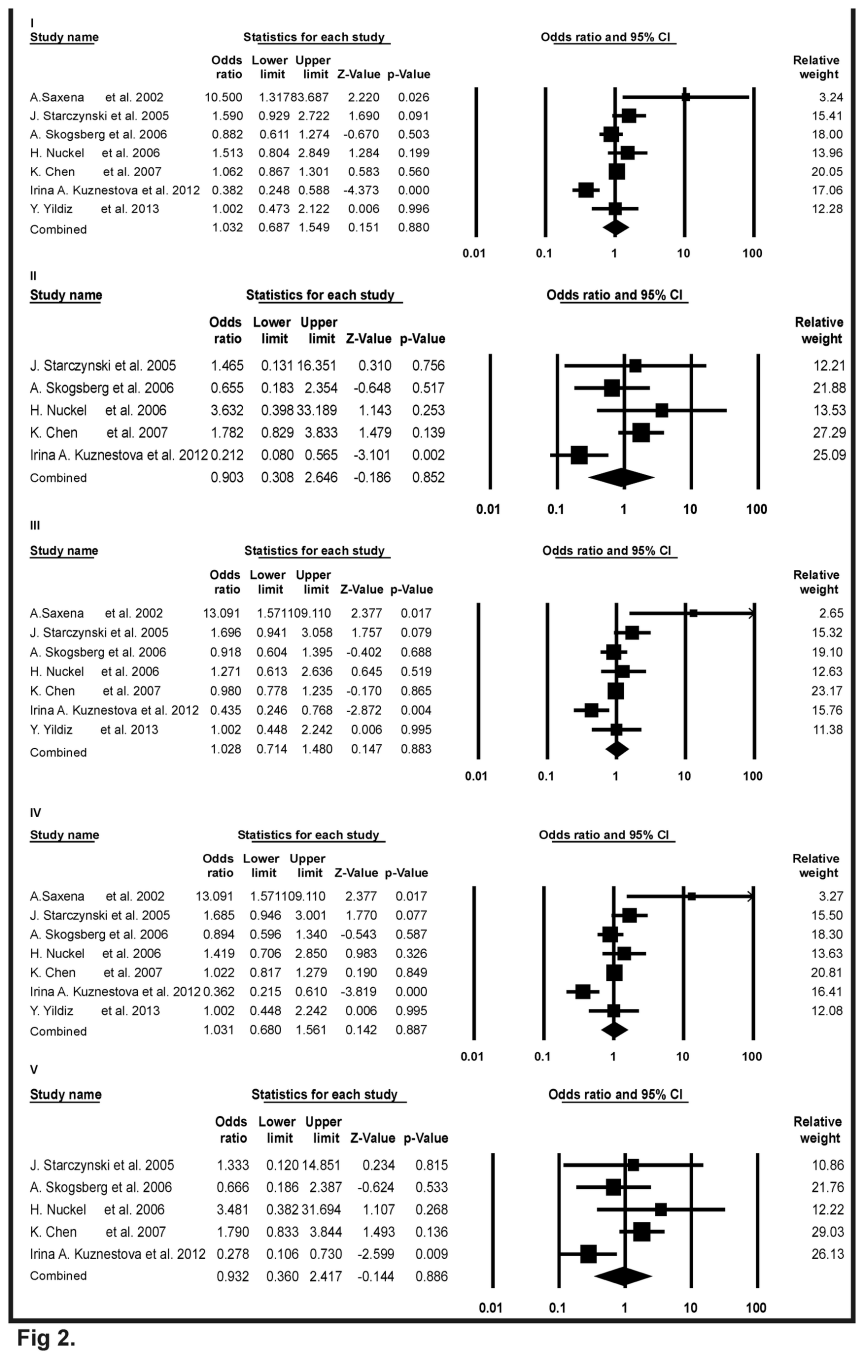
Forest plots of meta-analysis for Bax-248G>A polymorphism and cancer risk. The squares and horizontal lines correspond to the study specific odds ratios (ORs) and 95% confidence intervals (CI) respectively. The area of the squares reflects the study specific weight (inverse of the variance). The diamond represents the pooled ORs and 95%CI.

First we assessed Hardy-Weinberg equilibrium (HWE) for each study using SNPalyze software (Dynacom, Japan) to identify systematic genotyping errors. A power calculation was carried out a priori using PS: Power and Sample Size Calculation Software (http://biostat.mc.vanderbilt.edu/twiki/bin/view/Main/PowerSampleSize) which showed that a sample size of nearly 200 controls and cases would provide a power of more than 50%. Genotype and allele frequency were calculated by manual counting. All statistical analysis for the current meta-analysis was performed by comprehensive meta-analysis version 2 software. The association of Bax-248GA polymorphism with cancer was assessed by calculation of odds ratios (ORs) and 95% confidence intervals (CIs) in various models: (i) the allele contrast (A versus G), (ii) homozygous comparison (AA versus GG), (iii) heterozygous comparison (GA versus GG), (iv) dominant model (AA+GA versus GG) and (v) recessive model (AA versus GG+GA) in study I, II, III, IV and V respectively. Also, the Z and p-values were calculated in forest plot (Figure 2.) In all cases the results of the heterogeneity test were P_heterogeneity_ < 0.05, so ORs were pooled according to the random effects model (the Der Simonian and Laird method). In addition, I^2^ statistics was used to quantify inter study variability that can be attributed to heterogeneity rather than chance. It ranges between 0% and 100%, where a value of 0% indicates no observed heterogeneity and larger values indicate an increasing degree of heterogeneity (I^2^ = 0–25%, no heterogeneity; I^2^ = 25–50%, moderate heterogeneity; I^2^ = 50–75%, large heterogeneity; I^2^=75–100%, extreme heterogeneity). Moderate heterogeneity was observed in most of the models which were included for the analysis (Overall allele, A vs. G: P_heterogeneity_ < 0.0001, I^2^ = 79.42; homozygous comparison, AA vs. GG: P_heterogeneity_ = 0.01, I^2^ = 69.92; heterozygous comparison, GA vs. GG: P_heterogeneity_ = 0.008, I^2^ = 65.72; Overall dominant model, AA+GG vs. GG: P_heterogeneity_ < 0.0001, I^2^= 75.54; Overall recessive model, AA+GG vs. GG: P_heterogeneity_ = 0.03, I^2^= 62.07). 

## Results

### Study Characteristic

We identified 230 potentially relevant articles from our search of the published literature, of which 196 articles were excluded after initial review. Thus, a total of 34 studies were identified through the selection based on inclusion criteria. During extraction of data 25 articles that were not relevant to Bax-248GA polymorphism and cancers were excluded. Finally, again two studies were excluded after full text review, one due to lack of proper categorization of controls/ cases [33] and another is not providing relevant data [38]. Therefore, seven case-control studies including 1772 cases and 1708 controls (updated in March, 2013) were identified and included in the final meta-analysis (Figure 1.) The main characteristics were presented in Table 1. Sample size of the seven studies ranged from 59 to 1748. The seven studies include population from the United States of America (USA), United Kingdom (UK), Germany, Canada, Sweden, Russia, Turkey and Finland [30–32,34–37]. The distribution of the Bax-248GA polymorphism frequency among cases and controls of the seven studies in different cancer types (CLL, squamous cell carcinoma, lung cancer and breast cancer) were listed in Table 1. The genotype frequency in controls of all the studies are obeying the HWE except the study of Kuznestova et al [32].

**Table 1:** Distribution of Bax-248G>A genotype among cancer cases and controls includes in meta-analysis.

**First author, Year, Ref**	Country	Cancer type	Genotyping method	Sample size	Controls			Cases			*p* value of HWE	Power (%)
				**(Control/Case)**	**GG**	**GA**	**AA**	**GG**	**GA**	**AA**		
**A.Saxena, 2002**, [30]	Canada	Chronic lymphocytic leukemia	PCR	25/34	24	1	0	22	12	0	0.91	**17**
**J. Starczynski, 2005**, [33]	UK	Chronic lymphocytic leukemia	PCR	135/203	115	19	1	157	44	2	0.82	82
**A. Skogsberg, 2006**, [34]	Sweden, Germeny, Finland	Chronic lymphocytic leukemia	PCR	207/463	163	40	4	373	84	6	0.40	99
**H. Nuckel, 2006**, [35]	Germany	Chronic lymphocytic leukemia	PCR	95/112	79	15	1	87	21	4	0.76	58
**K. Chen, 2007**, [36]	USA	Squamous cell carcinoma	PIRA-PCR	934/814	723	200	11	627	170	17	0.49	100
**Irina A. Kuznetsova, 2012**, [32]	Russia	Lung Cancer	PCR-RFLP	230/93	111	80	39	67	21	5	0.0005	80
**Yemilha Yildiz, 2013**, [37]	Turkey	Breast cancer	PCR	82/53	63	19	0	43	13	0	0.23	39

NOTE : PCR; polymerase chain reaction, PIRA; Primer-introduced restriction analysis, HWE; Hardy-Weinberg equilibrium.

### Meta-analysis results

In total, seven independent studies were investigated and their results were meta-analyzed. Forest plots in Figure 2, showed the detail results of the Bax-248GA polymorphism and risk of cancer. Overall analysis were observed for all genetic models (OR = 1.032, 95% CI = 0.687 to 1.549, Z-value = 0.151, p-value = 0.880 for A versus G in study I; OR = 0.903, 95% CI = 0.308 to 2.646, Z-value = -0.186, p-value = 0.852 for AA versus GG in study II; OR = 1.028, 95% CI = 0.714 to 1.480, Z-value = 0.147, p-value = 0.883 for GA versus GG in study III; OR=1.031, 95% CI = 0.680 to 1.561, Z-value = 0.142, p-value = 0.887 for dominant model AA+GA versus GG in study IV; OR=0.932, 95% CI= 0.360 to 2.417, Z-value= -0.144, p-value=0.886 for recessive model AA versus GG+GA in study V). In all cases, p-values were > 0.05. So none of the model showed any significant associations for Bax-248GA polymorphism and risk of cancer.

### Sensitivity Analyses

Sensitivity analyses were performed to assess the stability of the results by sequential omission of individual study each time. This reflects the influence of individual data set to the pooled ORs. The significance of pooled ORs was not influenced excessively by omitting any single study (supplementary figures: S1, S2, S3, S4, S5, S6 and S7).

### Publication Bias

**Figure 3:**
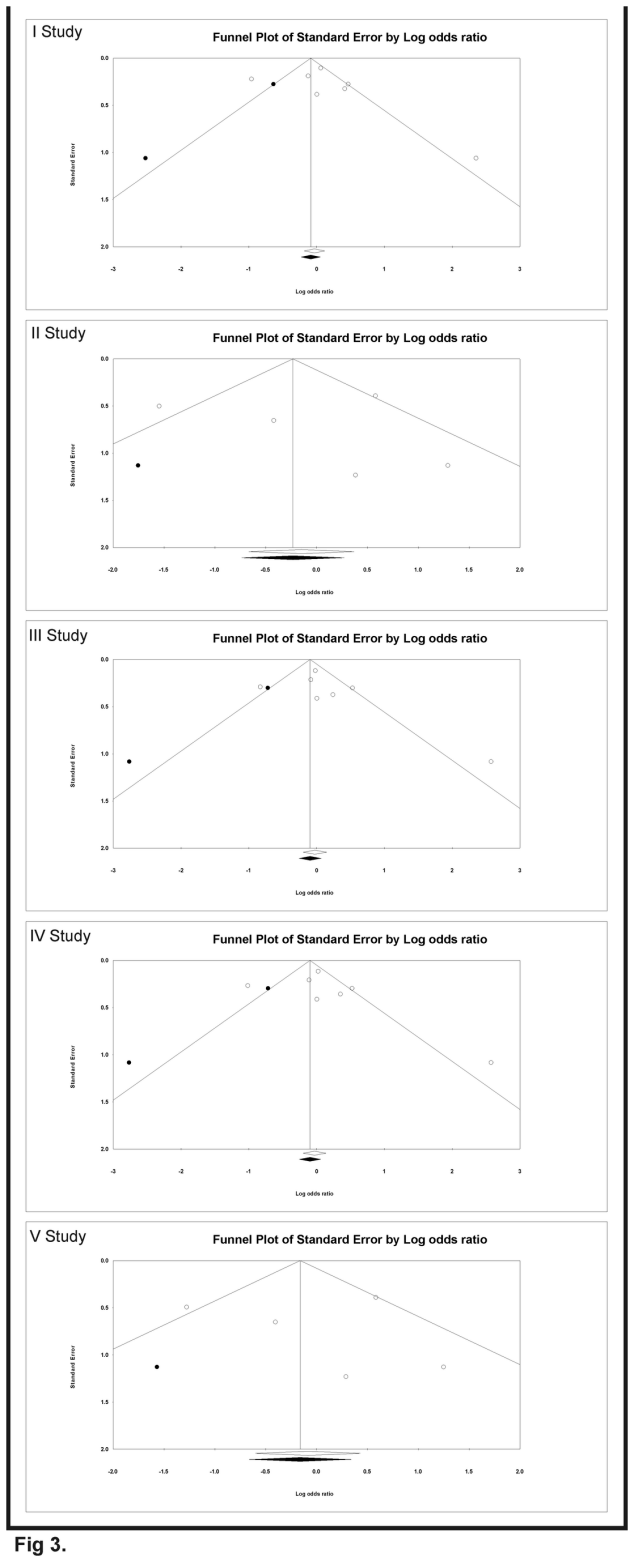
Funnel plots of the Egger’s test to detect publication bias. Each point represents a separate study. The OR was plotted on a logarithmic scale against the precision (the reciprocal of the SE) of each study.

Begg’s funnel plot and Egger’s linear regression tests [39] were conducted to assess the publication bias in the reports included for meta-analysis. The funnel plot is a plot of a measure of study size (usually standard error or precision) on the vertical axis as a function of effect size on the horizontal axis. The shape of funnel plots did not reveal any evidence of asymmetry in all of the comparison models: (i) the allele contrast (A versus G), (ii) homozygous comparison (AA versus GG), (iii) heterozygous comparison (GA versus GG), (iv) dominant model (AA+GA versus GG) and (v) recessive model (AA versus GG+GA) in study I, II, III, IV and V respectively (Figure 3.). Then, Egger’s test was used to provide statistical evidence of funnel plot symmetry. This test assesses the bias by using precision to predict the standardized effect. In this present analyses for study I: intercept = 0.91, 95% CI = -3.51 to 5.35, P-value = 0.61, SE = 1.72, t-value = 0.53, df = 5; For study II: intercept = 0.45, 95% CI = -7.13 to 8.05, P-value =0.86, SE = 2.38, t-value = 0.19, df = 3; For study III: intercept = 1.09, 95% CI = -2.21 to 4.40, P-value =0.43, SE = 1.28, t-value = 0.85, df = 5; For study IV: intercept = 0.97, 95% CI = -3.06 to 5.02, P-value = 0.56, SE = 1.57, t-value = 0.62, df = 5; For study V: intercept = 0.36, 95% CI = -6.39 to 7.11, P-value = 0.87, SE = 2.12, t-value = 0.17, df = 3. The results did not show any evidence of publication bias (Table 2.).

**Table 2:** Statistics to test publication bias and heterogeneity in meta-analysis for Bax -248G>A polymorphism and cancer risk.

**Comparisons**	**Egger’s regression analysis**							**Heterogeneity analysis**		**Model used for meta-analysis**
	**Intercept**	**95% Confidence Interval**	**P- value**	**SE**	**t-value**	**df**		**P_heterogeneity_**	**I^2^ (%)**	
**A vs G**	0.91	-3.51 to 5.35	0.61	1.72	0.53	5		<0.0001	74.92	Random
**AA vs GG**	0.45	-7.13 to 8.05	0.86	2.38	0.19	3		0.01	69.92	Random
**GA vs GG**	1.09	-2.21 to 4.40	0.43	1.28	0.85	5		0.008	65.72	Random
**AA+GA vs GG**	0.97	-3.06 to 5.02	0.56	1.57	0.62	5		<0.0001	75.54	Random
**AA vs GG+GA**	0.36	-6.39 to 7.11	0.87	2.12	0.17	3		0.03	62.07	Random

## Discussion

Apoptosis is an event that leads to the death of cell without releasing harmful substances into the tissue. In mitochondria mediated apoptotic cell death process Bax acts as an essential gatekeeper as its activation irreversibly commits the most of the cells to die [40,41]. It has been studied extensively on its relationship with different types of cancer, such as  pancreatic [19,42], bladder [21], gastric [18], colorectal [43,44], esophageal [16], lung [32,45,46], cervical [47], colon [48,49], prostate carcinoma [50,51], squamous cell carcinoma of the head and neck [36], nasopharyngeal carcinoma [52], breast carcinomas [32,53], ovarian carcinoma [54], renal and transitional cell cancer [55], gliomas [56], CLL [30,31,33–35,38,57], Hodgkin’s lymphoma [17], non-Hodgkin's lymphoma [58], myeloma [59], acute leukemia [60], etc. Bax promoter contains response elements for an important tumor suppressor p53 and this affects gene expression [28]. With respect to the important roles of Bax in apoptosis, it is biologically plausible that its polymorphism may modulate the risk of cancer. Altered expression of Bax protein seems relevant to carcinogenesis, but mutations leading to the deregulation and correlations of these mutations with cancer have attracted the attention among researcher. Several groups have investigated the relations between Bax-248GA polymorphism and cancer in different studies so far. In the initial report by Saxena et al. during 2002, in a study of 25 controls and 34 CLL patients they have shown that an increased genotype frequency of the Bax-248GA polymorphism in patient compared to controls, particularly in higher disease stage. In this study, genotype frequency in the control group was 5.7% for GA genotypes, which was 68.7 % for CLL in stage I-IV [30]. This finding was supported by a study of Moshynska et al. in which they have demonstrated this SNP as a cause of reduced Bax protein expression [31], which may be a reason of drug resistance by CLL patient. In a recent study by Kuznestova et al. in 2012, showed a significant increase in the frequency of -248G allele (88.33%) and -248GG genotype (72.04%) in lung cancer patients which were 65.65% and 48.26% respectively for healthy controls. Thus, they have shown the protective role of this polymorphism. 

However, the results from other groups reported lack of association of this polymorphism and risk of cancer. Starczynski et al. in their study found 23% of CLL and 15% of control with no significant allele frequency between the two groups [33]. Skosberg et al. in their CLL patient found no significant difference in overall survival with or without the Bax polymorphism [34]. Nuckel et al. showed genotype distribution between CLL patients (87 GG, 21AG, 4 AA) and healthy controls (79 GG, 15 AG, 1 AA) were not significantly different suggesting that this polymorphism may not increase the susceptibility for CLL (G-allele frequency CLL patients: 0.87; controls: 0.91) [35]. Chen et al. did not find any statistically significant difference in the frequency distributions of the Bax-248 G>A SNP between cases and controls (P = 0.625). When the Bax GG genotype was taken as the reference group, no association was found between the AA and AG variant genotypes with squamous cell carcinoma of the head and neck (SCCHN) risk [36]. Yildiz et al. showed that Bax Bax-248GA genotype and allele frequency between controls and breast cancer patients were not statistically significant (p = 0.866, p = 0.856 respectively) [37]. On the whole, the results about the association between Bax-248GA polymorphism and cancer risk remains conflicting and inconclusive. The conflicting results are possibly because of a small effect of the Bax-248GA polymorphism on cancer risk or the relatively low statistical power of published studies. So, this meta-analysis was needed to show a quantitative approach for combining the different available results. Meta-analysis is a powerful method which can combine the findings of several independent similar studies with inconsistent results to produce a single estimate of the major effect with enhanced precision [39]. In this present meta-analysis, a total of seven case-control studies were analyzed to provide a comprehensive assessment of the association between the Bax-248GA polymorphism and overall cancer risk. The studies involved in this meta-analysis were relatively small, but the number of total controls and cases were substantial, which increased the statistical power of the analysis. The case-control studies included in this analysis were satisfactory as they met our preset inclusion criteria. We didn’t detect any publication bias suggesting that the whole pooled results were unbiased.

Odds ratios were used to determine the relative odds of the occurrence of the cancer risk with Bax-248GA polymorphism and 95% CI to estimate the precision of the OR. A large CI indicates a low-level of precision of the OR, whereas a small CI indicates a higher precision of the OR. In our results as shown in Figure 2, individuals with allele A had an increased risk of cancer compared with wild type allele A (OR = 1.032, 95% C I= 0.687 to 1.549). Individuals with homozygous variant AA had a decreased risk of cancer compared with wild type GG variant (OR = 0.903, 95% CI = 0.308 to 2.646) and with heterozygous variant GA had an increased risk of cancer compared with wild type GG variant (OR = 1.028, 95% CI = 0.714 to 1.480). In dominant model, individuals with genotype (AA+GA) had an increased risk of cancer compared with wild type GG variant (OR = 1.031, 95% CI = 0.680 to 1.561) and in recessive model, homozygous variant AA had a decreased risk of cancer compared with (GA+GG) genotype (OR = 0.932, 95% CI = 0.360 to 2.417). But in the entire above five comparison model p-values were > 0.05 which suggested a lack of significant association towards increase or decrease risk of cancer due to Bax-248GA polymorphism.

Heterogeneity was observed among these studies through I^2^ value. The heterogeneity may be due to various factors, such as diversity in population characteristic, study design, differences in the number of cases and controls, genotype method, etc. Some eligible unpublished publications were not available to include in the present meta-analysis, which might affect the results. Selection bias could have played a role to influence the result because the genotype distribution of this polymorphism deviated from HWE in one study [32]. The results were based on unadjusted estimates, while a more precise statistical analysis should be conducted if individual dataset were available. This would allow the investigator for adjustment by other confounding variables including environmental factors and other lifestyle.

To the best of our knowledge this is the first meta-analysis regarding the comprehensive assessment of the relationship between Bax-248GA polymorphism and the risk of cancer. Our results did not support a genetic association between this polymorphism and susceptibility to cancer corroborates with some of the previous case-control studies [34–37]. Neither allele frequency nor genotype distributions were significantly associated with susceptibility to cancer which hypotheses that Bax-248GA polymorphism may have no role in cancer vulnerability. In addition, because of the relatively small sample size, the result needed further validation and confirmation with large sample studies.

Following practical recommendations which constitute action points may be consider in future association studies:

The lack association in this study of Bax-248GA polymorphism and cancer risk should be replicated with biological functions to better motivate the study and to enable interpretation of results. Histopathological and clinical data can sub classify the type and stage of many cancer cases to get more homogeneous population for analysis.

Try to decrease false positive and negative results by conducting the studies in a large sample with stratification by age, sex, food habit, lifestyle and ethnicity.

Studies examining the combined effects of different Bax polymorphism or different polymorphisms of Bax related genes (e.g. Bcl2) should be investigated.

The role of environmental factors and epistatic interaction are not considered in this study due to lack of information in original published articles which need to explore further to draw more reliable conclusions.

## Conclusion

This study reveals no significant association of Bax-248GA SNP to cancer risk as evidenced from all genetic models. Moreover, it would be enlightening to extend the investigation to a wider range of human populations including different cancer types which may lead to a better, comprehensive understanding of the association between this polymorphism and susceptibility to cancer. The ability to implement the involvement of this SNP towards cancer etiology and an individual’s chance for developing cancer by considering the proposed recommendation will help in the future. This may further translate into basic research or clinical practice to save the human lives.

## Supporting Information

Checklist S1 **PRISMA checklist.**
(DOC)

Figure S1 **Forest plot (**A**) and funnel plot (**B**) of Bax-248G>A polymorphism in association with cancers after omission of A.** **Saxena et al. (2002) study**.
In forest plot (A), the squares and horizontal lines correspond to the study specific odds ratios (ORs) and 95% confidence intervals (CI) respectively. The area of the squares reflects the study specific weight (inverse of the variance). The diamond represents the pooled ORs and 95%CI. In funnel plot (B), each point represents a separate study. The OR was plotted on a logarithmic scale against the precision (the reciprocal of the SE) of each study.
(TIF)

Figure S2 **Forest plot (**A**) and funnel plot (**B**) of Bax-248G>A polymorphism in association with cancers after omission of J.** **Starczynski et al. (2005) study**.
In forest plot (A), the squares and horizontal lines correspond to the study specific odds ratios (ORs) and 95% confidence intervals (CI) respectively. The area of the squares reflects the study specific weight (inverse of the variance). The diamond represents the pooled ORs and 95%CI. In funnel plot (B), each point represents a separate study. The OR was plotted on a logarithmic scale against the precision (the reciprocal of the SE) of each study.
(TIF)

Figure S3 **Forest plot (**A**) and funnel plot (**B**) of Bax-248G>A polymorphism in association with cancers after omission of A.** **Skogsberg et al. (2006) study**.
In forest plot (A), the squares and horizontal lines correspond to the study specific odds ratios (ORs) and 95% confidence intervals (CI) respectively. The area of the squares reflects the study specific weight (inverse of the variance). The diamond represents the pooled ORs and 95%CI. In funnel plot (B), each point represents a separate study. The OR was plotted on a logarithmic scale against the precision (the reciprocal of the SE) of each study.
(TIF)

Figure S4 **Forest plot (**A**) and funnel plot (**B**) of Bax-248G>A polymorphism in association with cancers after omission of H.** **Nuckel et al. (2006) study**.
In forest plot (A), the squares and horizontal lines correspond to the study specific odds ratios (ORs) and 95% confidence intervals (CI) respectively. The area of the squares reflects the study specific weight (inverse of the variance). The diamond represents the pooled ORs and 95%CI. In funnel plot (B), each point represents a separate study. The OR was plotted on a logarithmic scale against the precision (the reciprocal of the SE) of each study.
(TIF)

Figure S5 **Forest plot (**A**) and funnel plot (**B**) of Bax-248G>A polymorphism in association with cancers after omission of K.** **Chen et al. (2007) study**.
In forest plot (A), the squares and horizontal lines correspond to the study specific odds ratios (ORs) and 95% confidence intervals (CI) respectively. The area of the squares reflects the study specific weight (inverse of the variance). The diamond represents the pooled ORs and 95%CI. In funnel plot (B), each point represents a separate study. The OR was plotted on a logarithmic scale against the precision (the reciprocal of the SE) of each study.
(TIF)

Figure S6 **Forest plot (**A**) and funnel plot (**B**) of Bax-248G>A polymorphism in association with cancers after omission of Irina A.** **Kuznetsova et al. (2012) study**.
In forest plot (A), the squares and horizontal lines correspond to the study specific odds ratios (ORs) and 95% confidence intervals (CI) respectively. The area of the squares reflects the study specific weight (inverse of the variance). The diamond represents the pooled ORs and 95%CI. In funnel plot (B), each point represents a separate study. The OR was plotted on a logarithmic scale against the precision (the reciprocal of the SE) of each study.
(TIF)

Figure S7 **Forest plot (**A**) and funnel plot (**B**) of Bax-248G>A polymorphism in association with cancers after omission of Yemilha Yildiz et al.** **(2013) study**.
In forest plot (A), the squares and horizontal lines correspond to the study specific odds ratios (ORs) and 95% confidence intervals (CI) respectively. The area of the squares reflects the study specific weight (inverse of the variance). The diamond represents the pooled ORs and 95%CI. In funnel plot (B), each point represents a separate study. The OR was plotted on a logarithmic scale against the precision (the reciprocal of the SE) of each study.
(TIF)
