# Supplementary figures and images for: Lack of Association between Bax Promoter (-248G>A) Single Nucleotide Polymorphism and Susceptibility towards Cancer: Evidence from a Meta-Analysis
Source: PLoS One. 2013 Oct 17;8(10):e77534. doi: 10.1371/journal.pone.0077534 (PMC3798304; doi:10.1371/journal.pone.0077534)

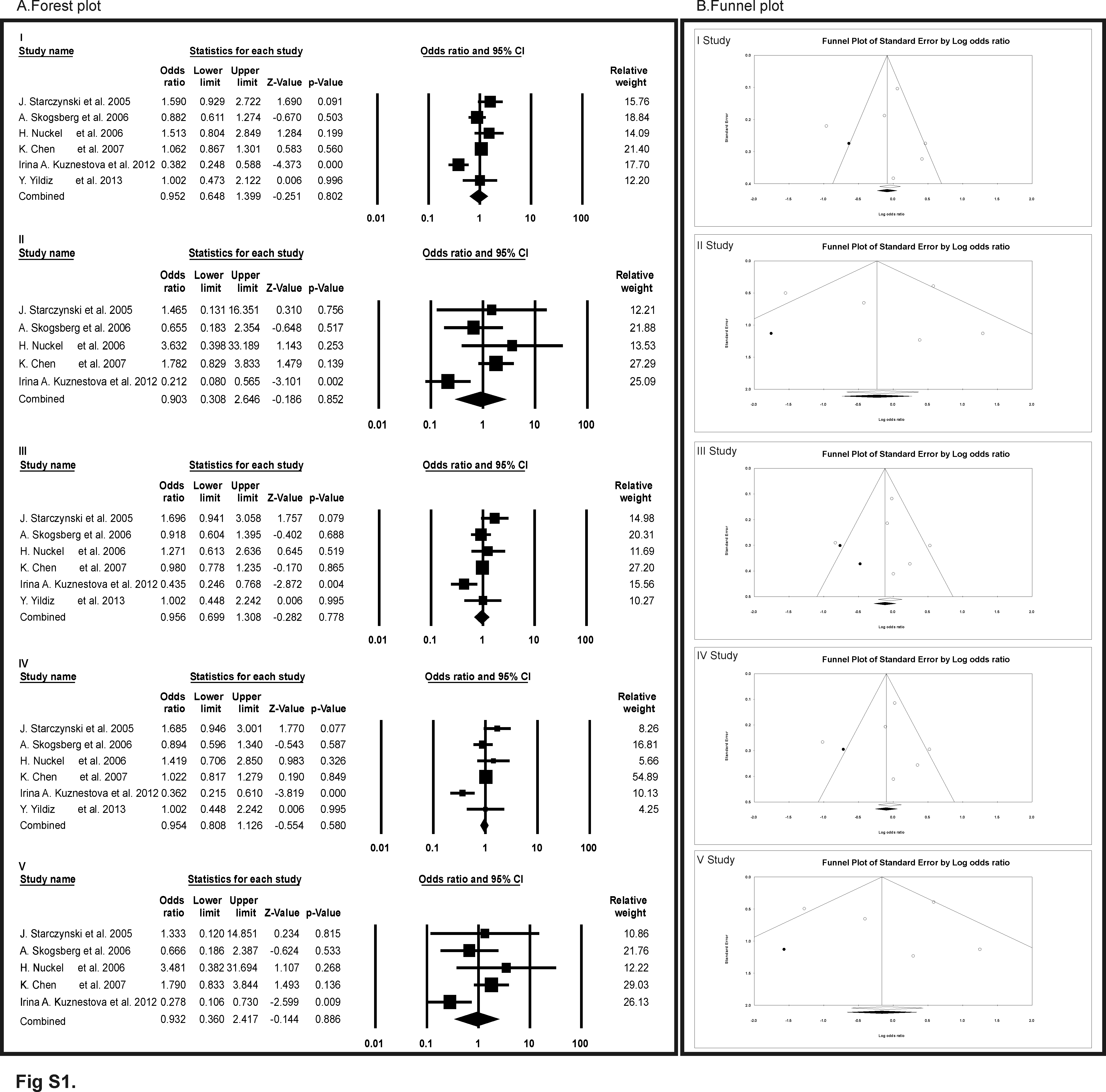

Supplement: Figure S1 — Forest plot (A) and funnel plot (B) of Bax-248G>A polymorphism in association with cancers after omission of A. Saxena et al. (2002) study. In forest plot (A), the squares and horizontal lines correspond to the study specific odds ratios (ORs) and 95% confidence intervals (CI) respectively. The area of the squares reflects the study specific weight (inverse of the variance). The diamond represents the pooled ORs and 95%CI. In funnel plot (B), each point represents a separate study. The OR was plotted on a logarithmic scale against the precision (the reciprocal of the SE) of each study. (TIF) [file pone.0077534.s002.tif]

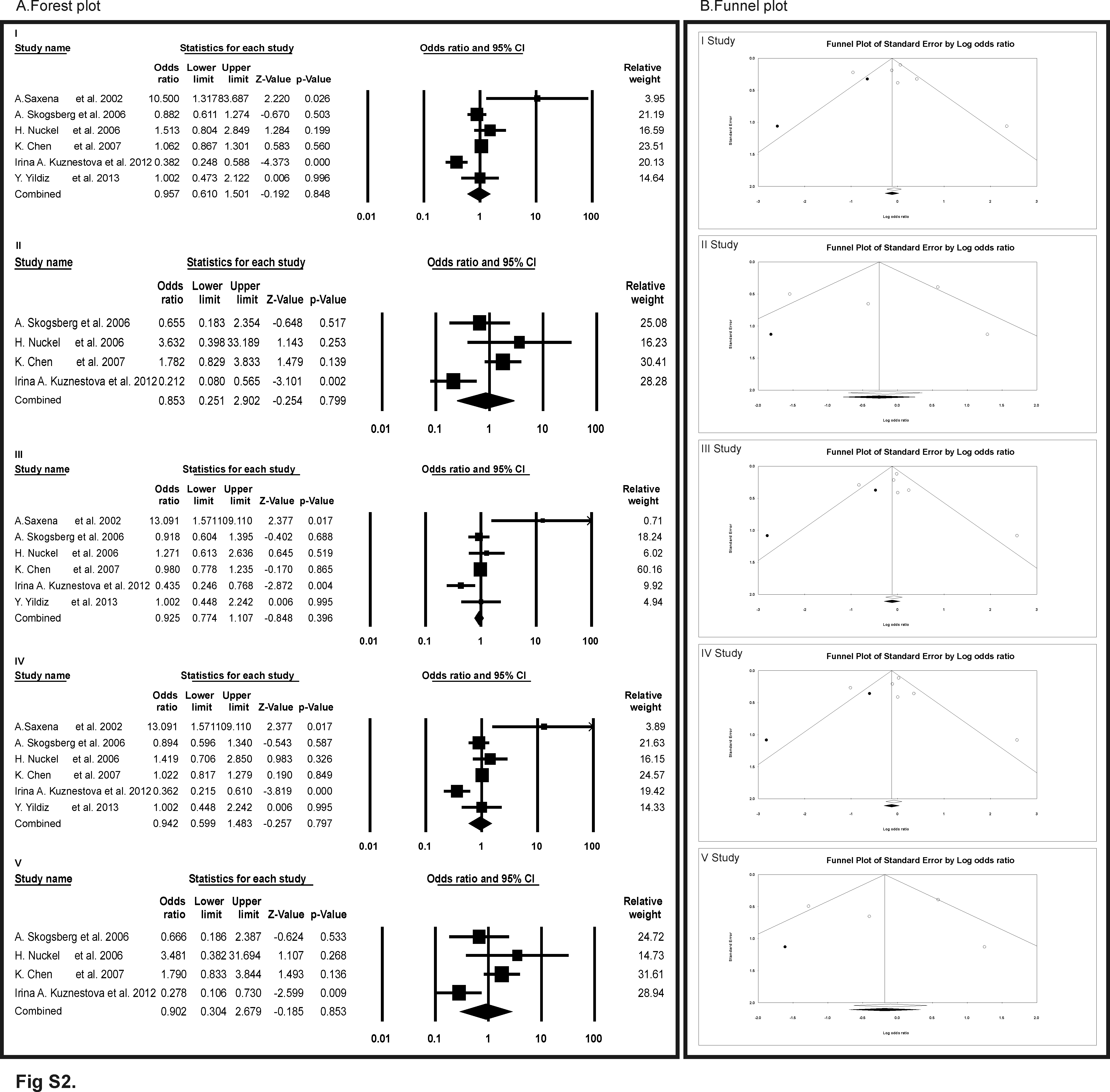

Supplement: Figure S2 — Forest plot (A) and funnel plot (B) of Bax-248G>A polymorphism in association with cancers after omission of J. Starczynski et al. (2005) study. In forest plot (A), the squares and horizontal lines correspond to the study specific odds ratios (ORs) and 95% confidence intervals (CI) respectively. The area of the squares reflects the study specific weight (inverse of the variance). The diamond represents the pooled ORs and 95%CI. In funnel plot (B), each point represents a separate study. The OR was plotted on a logarithmic scale against the precision (the reciprocal of the SE) of each study. (TIF) [file pone.0077534.s003.tif]

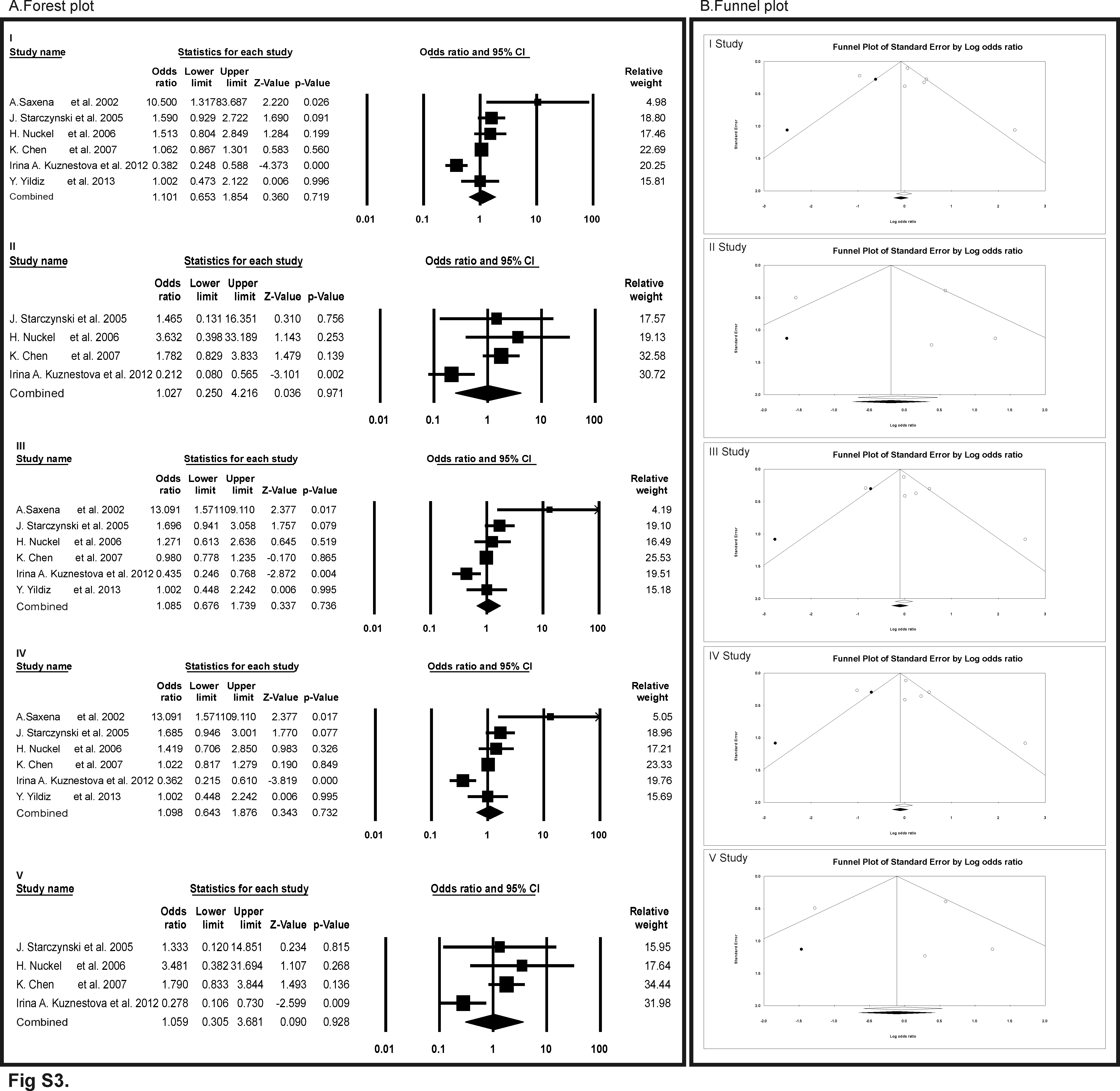

Supplement: Figure S3 — Forest plot (A) and funnel plot (B) of Bax-248G>A polymorphism in association with cancers after omission of A. Skogsberg et al. (2006) study. In forest plot (A), the squares and horizontal lines correspond to the study specific odds ratios (ORs) and 95% confidence intervals (CI) respectively. The area of the squares reflects the study specific weight (inverse of the variance). The diamond represents the pooled ORs and 95%CI. In funnel plot (B), each point represents a separate study. The OR was plotted on a logarithmic scale against the precision (the reciprocal of the SE) of each study. (TIF) [file pone.0077534.s004.tif]

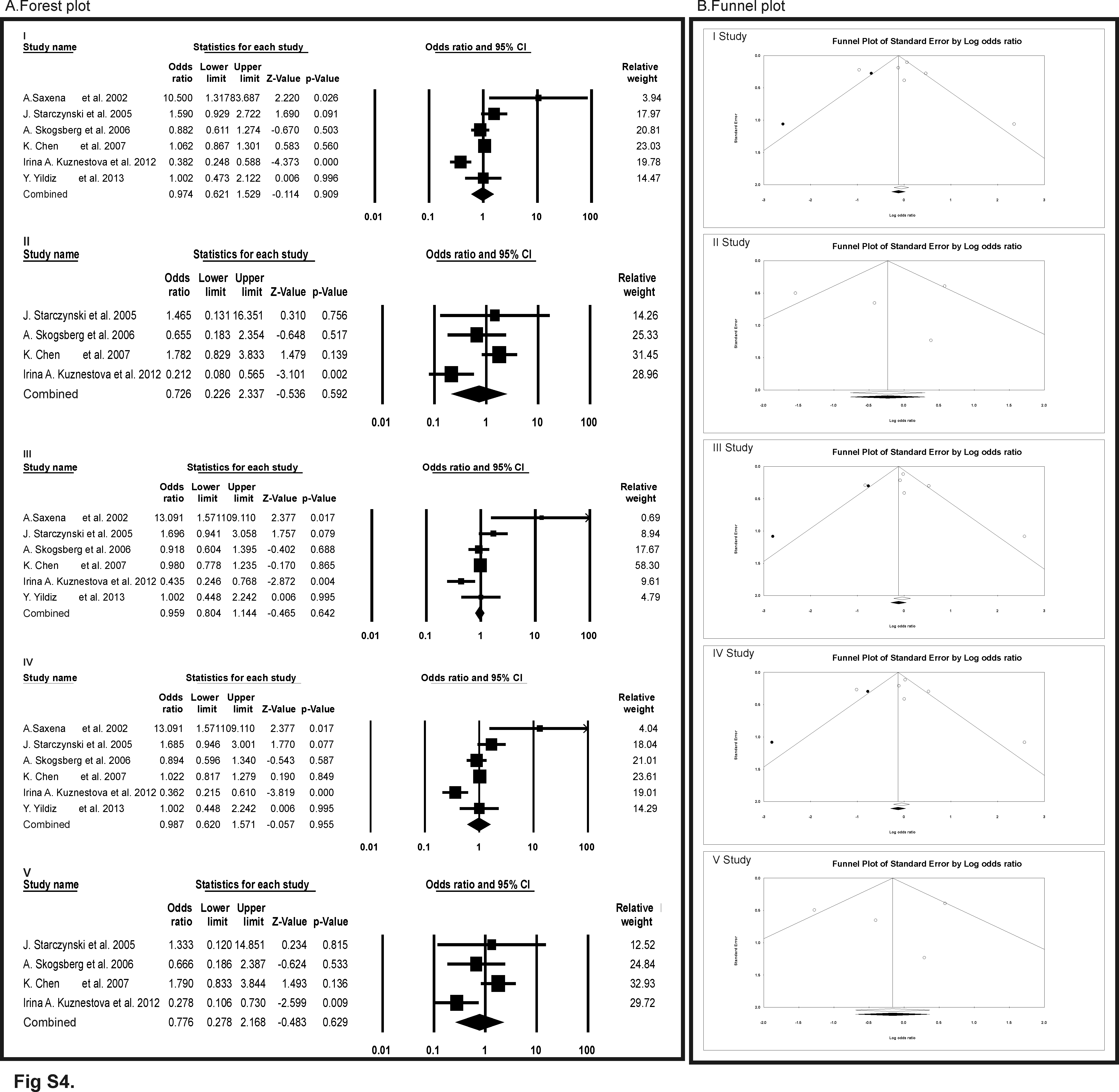

Supplement: Figure S4 — Forest plot (A) and funnel plot (B) of Bax-248G>A polymorphism in association with cancers after omission of H. Nuckel et al. (2006) study. In forest plot (A), the squares and horizontal lines correspond to the study specific odds ratios (ORs) and 95% confidence intervals (CI) respectively. The area of the squares reflects the study specific weight (inverse of the variance). The diamond represents the pooled ORs and 95%CI. In funnel plot (B), each point represents a separate study. The OR was plotted on a logarithmic scale against the precision (the reciprocal of the SE) of each study. (TIF) [file pone.0077534.s005.tif]

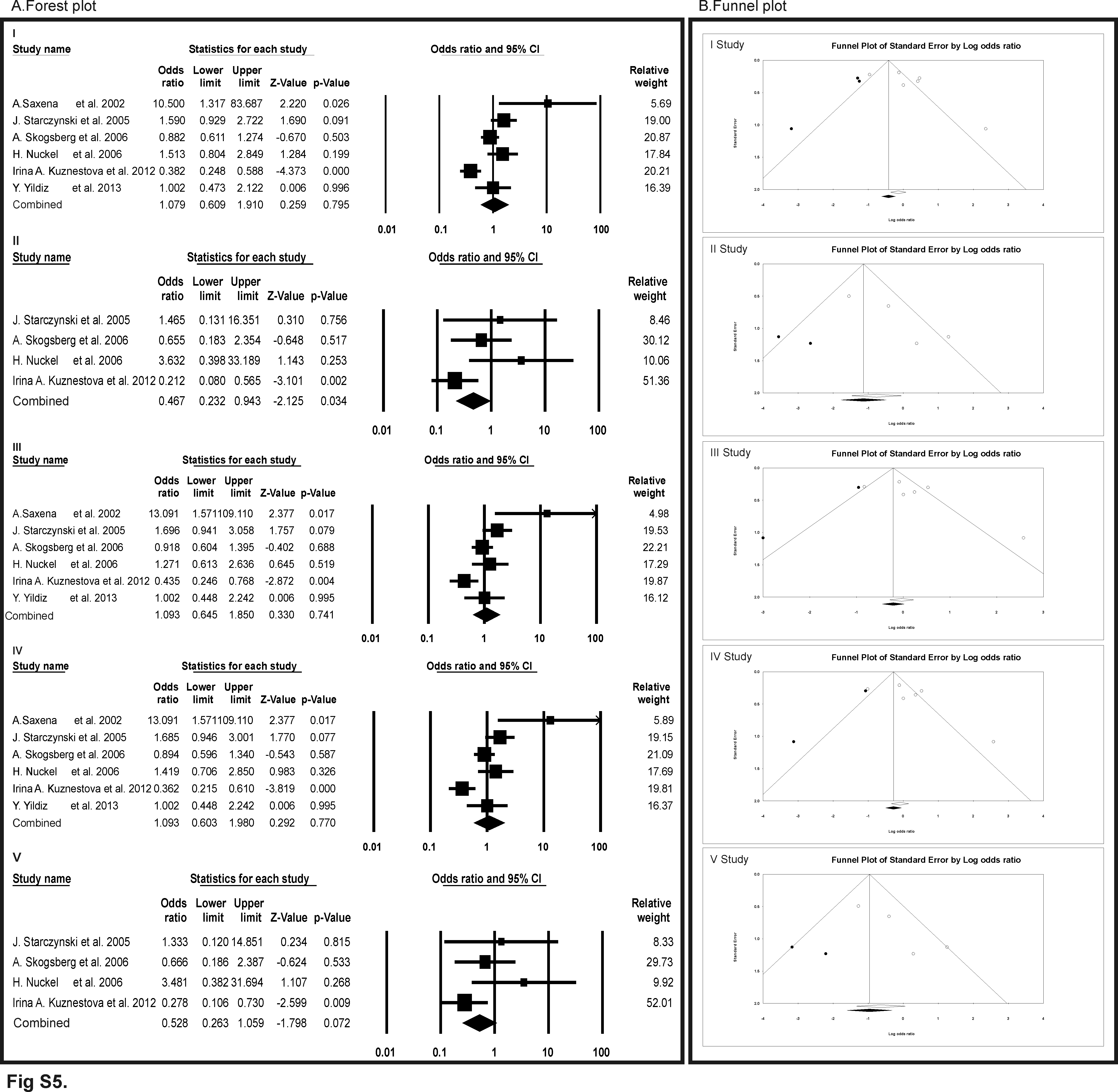

Supplement: Figure S5 — Forest plot (A) and funnel plot (B) of Bax-248G>A polymorphism in association with cancers after omission of K. Chen et al. (2007) study. In forest plot (A), the squares and horizontal lines correspond to the study specific odds ratios (ORs) and 95% confidence intervals (CI) respectively. The area of the squares reflects the study specific weight (inverse of the variance). The diamond represents the pooled ORs and 95%CI. In funnel plot (B), each point represents a separate study. The OR was plotted on a logarithmic scale against the precision (the reciprocal of the SE) of each study. (TIF) [file pone.0077534.s006.tif]

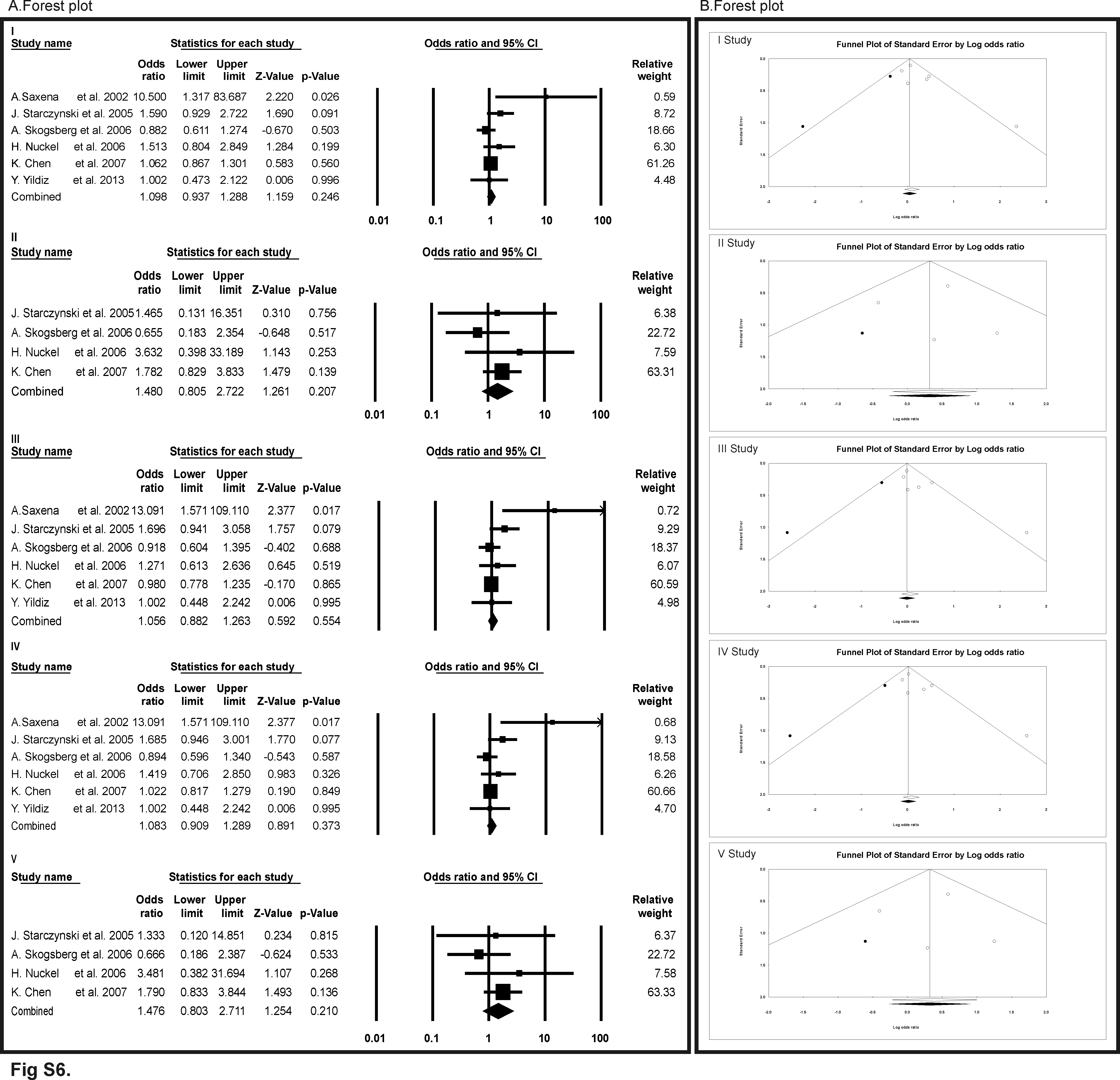

Supplement: Figure S6 — Forest plot (A) and funnel plot (B) of Bax-248G>A polymorphism in association with cancers after omission of Irina A. Kuznetsova et al. (2012) study. In forest plot (A), the squares and horizontal lines correspond to the study specific odds ratios (ORs) and 95% confidence intervals (CI) respectively. The area of the squares reflects the study specific weight (inverse of the variance). The diamond represents the pooled ORs and 95%CI. In funnel plot (B), each point represents a separate study. The OR was plotted on a logarithmic scale against the precision (the reciprocal of the SE) of each study. (TIF) [file pone.0077534.s007.tif]

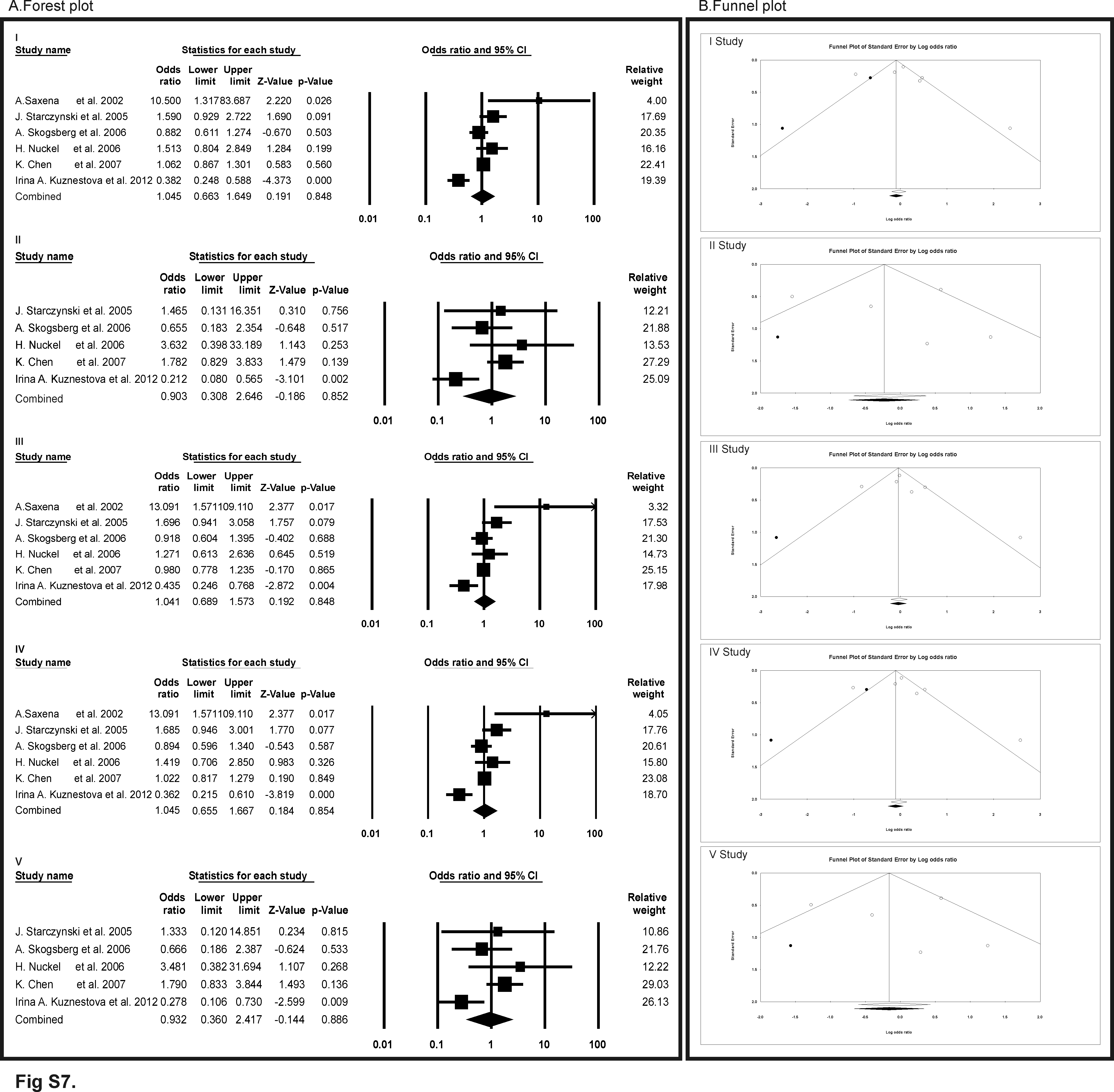

Supplement: Figure S7 — Forest plot (A) and funnel plot (B) of Bax-248G>A polymorphism in association with cancers after omission of Yemilha Yildiz et al. (2013) study. In forest plot (A), the squares and horizontal lines correspond to the study specific odds ratios (ORs) and 95% confidence intervals (CI) respectively. The area of the squares reflects the study specific weight (inverse of the variance). The diamond represents the pooled ORs and 95%CI. In funnel plot (B), each point represents a separate study. The OR was plotted on a logarithmic scale against the precision (the reciprocal of the SE) of each study. (TIF) [file pone.0077534.s008.tif]
